# Supplementary material for: An amino acid-resolution interactome for motile cilia illuminates the structure and function of ciliopathy protein complexes
Source: bioRxiv. 2023 Sep 21:2023.07.09.548259. Preprint. [Version 2] doi: 10.1101/2023.07.09.548259 (PMC10541116; doi:10.1101/2023.07.09.548259)
Supplement: 1 [file NIHPP2023.07.09.548259V2-supplement-1.pdf]

bioRxiv preprint doi: https://doi.org/10.1101/2023.07.09.548259; this version posted September 21, 2023. The copyright holder for this preprint (which was not certified by peer review) is the author/funder, who has granted bioRxiv a license to display the preprint in perpetuity. It is made available under aCC-BY 4.0 International license.

Supplementary Figures

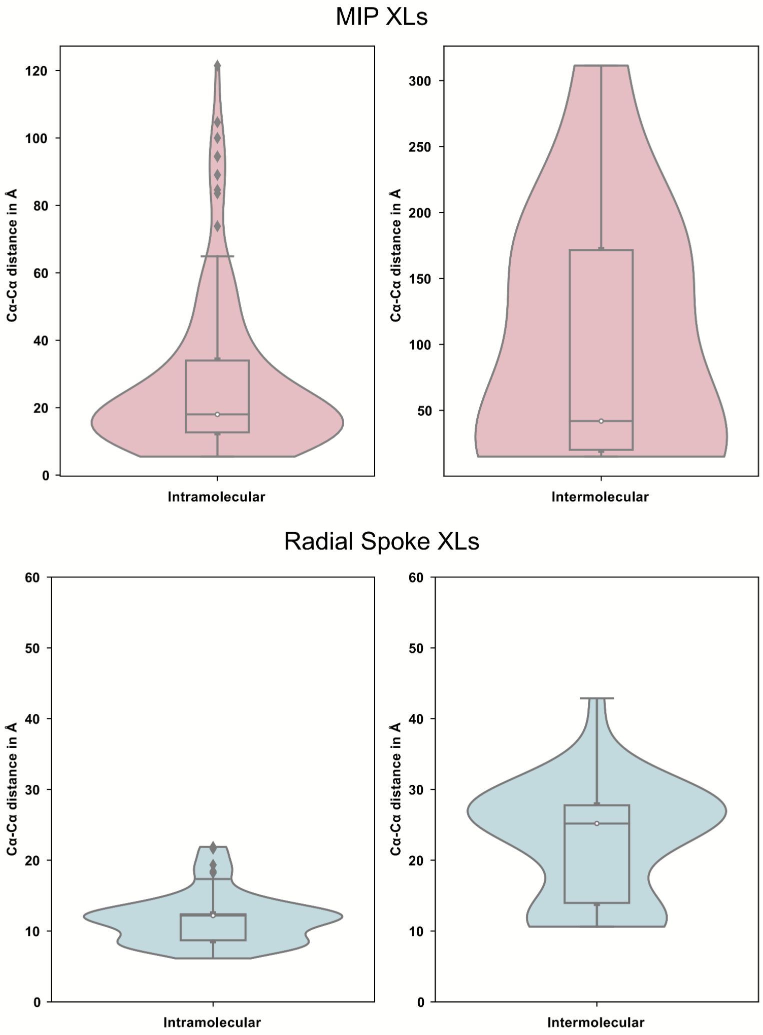

Supplementary Figure 1. Distance distribution of intra- and intermolecular cross-links of ciliary MIP and radial spoke proteins.

bioRxiv preprint doi: https://doi.org/10.1101/2023.07.09.548259; this version posted September 21, 2023. The copyright holder for this preprint (which was not certified by peer review) is the author/funder, who has granted bioRxiv a license to display the preprint in perpetuity. It is made available under aCC-BY 4.0 International license.

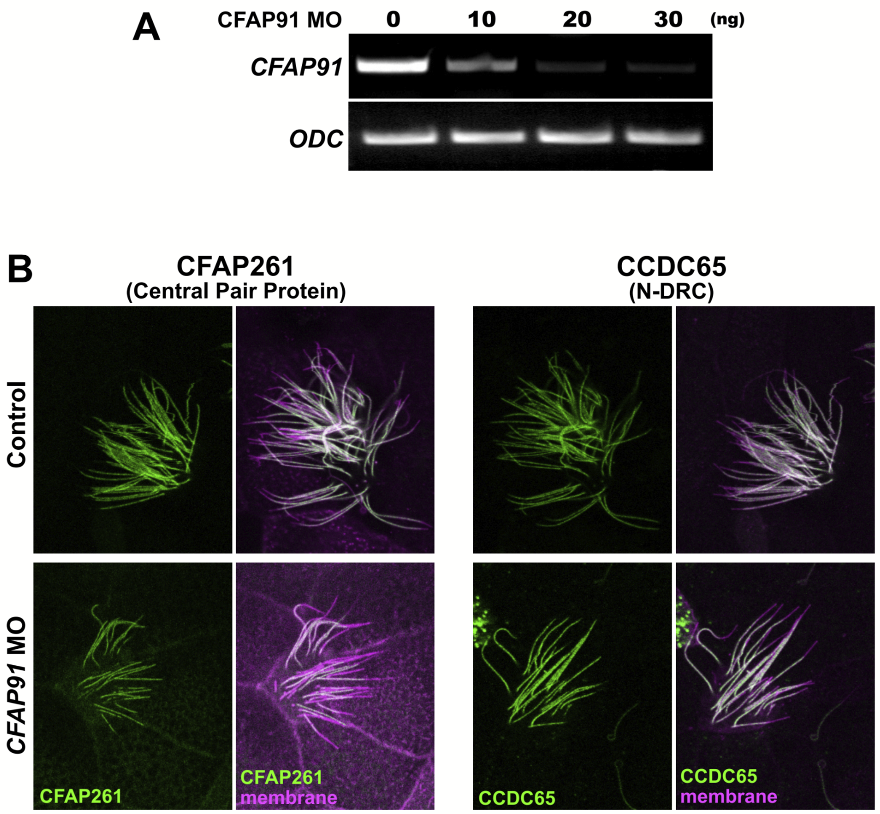

Supplementary Figure 2. (A) RT-PCR showed effective disruption of splicing by injection of *CFAP91* morpholino. **(B)** Localization of GFP fusions to indicated proteins in *Xenopus* MCCs.

bioRxiv preprint doi: https://doi.org/10.1101/2023.07.09.548259; this version posted September 21, 2023. The copyright holder for this preprint (which was not certified by peer review) is the author/funder, who has granted bioRxiv a license to display the preprint in perpetuity. It is made available under aCC-BY 4.0 International license.

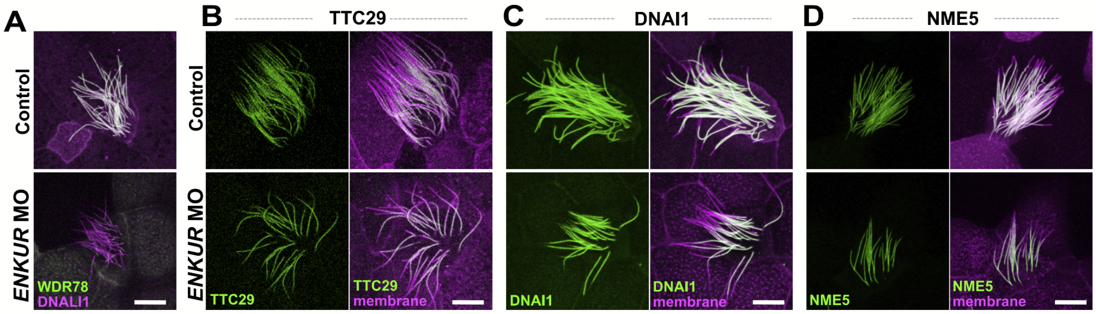

Supplementary Figure 3. (A) After ENKUR knockdown, GFP-WDR78 (an IDA-f subunit) failed to localize to the axoneme, while mCherry-DNALI1 remained unaffected. **(B)-(D)** The loss of *ENKUR* did not affect the localization of TTC29 (an IDA-d subunit), DNAI1 (an ODA subunit) and NME5 (a radial spoke protein) on the axonemes. Scale bars = 10 μm

Supplementary Table 1. List of unique cross-links with Tetrahymena and Uniprot gene identifiers, cross-linked amino acid positions, EggNOG (v. 5) orthogroup identifiers, and human orthologs.
